# Perioperative severe hemorrhage in pediatric kidney transplantation: frequency, risk factors, and outcomes

**DOI:** 10.1007/s00467-026-07391-7

**Published:** 2026-06-06

**Authors:** Marta L. A. Maia, Heitor P. Leite, Luciana S. Feltran, Maria Cristina Andrade, Maria F. C. Camargo, Paulo C. Koch Nogueira

**Affiliations:** 1 Department of Pediatrics, Universidade Federal de São Paulo – Escola Paulista de Medicina, Rua Botucatu, 740 _ Vila Clementino, São Paulo, SP 04023-062 Brazil; 2 Division of Pediatric Kidney Transplantation, São Paulo Higienopolis Samaritan Hospital, São Paulo, Brazil

**Keywords:** Kidney transplantation, Children, Severe hemorrhage, Incidence, Risk factors

## Abstract

**Background:**

To characterize severe hemorrhage and determine its frequency, risk factors, and consequences in the perioperative period of pediatric kidney transplantation.

**Methods:**

Single-center retrospective study of children undergoing their first kidney transplant between 2008 and 2015. Severe hemorrhage was defined using a data-driven cluster analysis to identify patients with high perioperative bleeding volume and blood component requirements. Additionally, severe hemorrhage was defined by the need for reoperation or administration of activated factor VII or fibrinogen for bleeding related to transplant. Thirty-eight exposure variables were examined, including clinical and laboratory data, donor and recipient characteristics, surgical technical variables, and medications. Analysis of hemorrhage consequences considered the occurrence of delayed renal function, hospitalization length during transplantation, estimated glomerular filtration rate post-transplantation, and five-year graft and patient survival.

**Results:**

Severe hemorrhage occurred in 16/218 cases (7.3%). In unadjusted logistic analyses, higher risk was observed among younger and smaller recipients, those receiving kidneys from older and living donors, and those exposed to a higher graft dose. Among laboratory markers, postoperative platelet count was associated with hemorrhage. Five-year graft survival was lower in children with severe hemorrhage compared with those without (62.5% vs. 91.5%, *p* < 0.001).

**Conclusion:**

The frequency of severe hemorrhage in children treated with kidney transplantation in our center was higher than reported in the literature. The type of donor and the combination of small recipients and large donors were the main risk factors. The need for a larger abdominal dissection area in small children receiving large grafts, contributing to the risk of bleeding, is a possible explanation for this finding.

**Graphical abstract:**

**Figure:**
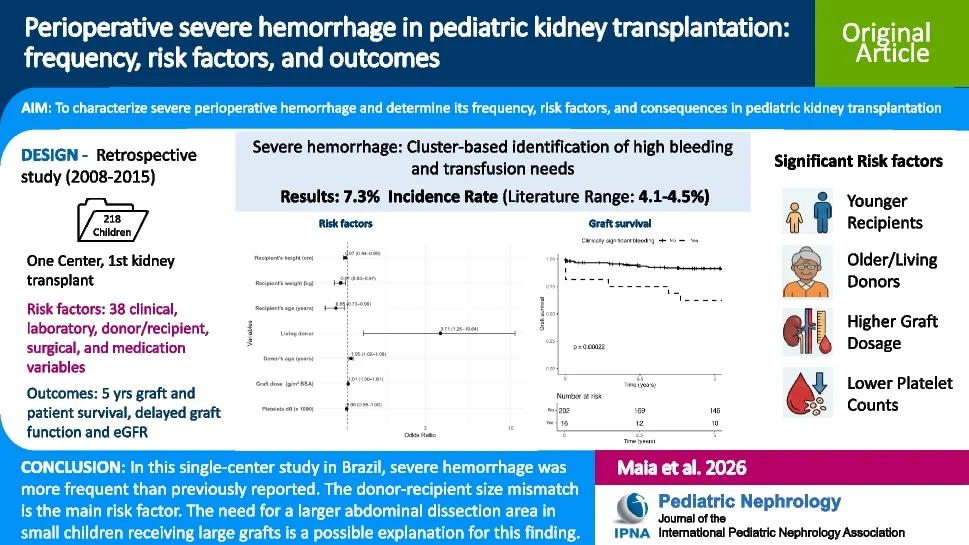
A higher resolution version of the Graphical abstract is available as Supplementary information

**Supplementary Information:**

The online version contains supplementary material available at https://doi.org/10.1007/s00467-026-07391-7.

## Introduction

Kidney transplantation is the preferred treatment for children with chronic kidney failure because it is associated with lower morbidity and mortality rates, better quality of life, and reduced treatment costs compared to dialysis methods [1–3]. However, a wide variety of complications can occur during the perioperative period, including hemodynamic instability, metabolic disorders, vascular thrombosis, and bleeding. Hemorrhagic complications may occur during the surgical procedure or shortly after transplantation, potentially causing significant morbidity [4–6].

Bleeding in pediatric kidney transplant recipients has been poorly described in the literature, with limited reports on risk factors and associated characteristics [7, 8]. In adults, the incidence can range from 0.2% to 14% [9], which may be attributed to the unique coagulation challenges seen in patients with chronic kidney disease (CKD) [10]. In children, Beetz et al. [7] retrospectively evaluated 221 kidney transplants, with an average follow-up period of 84 months. Hemorrhage was observed in ten cases (4.5%), typically occurring in the first five postoperative days. Hemorrhagic complications were defined as those requiring intraoperative or postoperative revision during primary hospitalization after kidney transplantation [7]. In Brazil, a retrospective study by Beatrice et al. involving 96 children who underwent kidney transplantation reported hemorrhagic complications in 13 patients. Of these, four (4.2%) had severe bleeding, two (2.1%) had moderate bleeding, and seven (7.3%) had mild bleeding [8].

The International Society on Thrombosis and Haemostasis (ISTH) defines major surgical bleeding as any of the following: fatal bleeding, symptomatic bleeding in a critical area or organ, extra-surgical-site bleeding with a drop in hemoglobin (Hb) of > 2 g/dL or the need for transfusion of > 2 units of whole blood or red blood cells, bleeding requiring reoperation, bleeding associated with hemodynamic instability, or a drop in Hb > 2 g/dL within 24 h of the bleeding event [11]. This standardization, introduced in 2010, addresses the variability in bleeding definitions across different studies. The ISTH definition was used as the main reference framework for major surgical bleeding, although it may not be fully applicable to the specific context of pediatric kidney transplantation. Although widely used to define major bleeding, this classification was developed for adults and has not been validated in patients with kidney disease. In pediatric kidney transplantation, recipients usually have some degree of anemia due to CKD and often receive red blood cell concentrates and volume infusions (colloids or crystalloids) to maintain graft perfusion. As a result, the ISTH criteria are not readily applicable to this clinical setting [11].

Little is known about the reasons why some patients with CKD develop hemorrhagic events whereas others are prone to thrombi formation. Studies have shown that CKD is a risk factor for thromboembolic events; however, patients with kidney failure also have an increased bleeding tendency due to alterations in both procoagulant and anticoagulant pathways. This hemostatic paradox is reflected by a reported incidence of clinically significant post-transplant bleeding of approximately 4%, compared with about 1% in general surgical populations [12].

Hemorrhagic events in the perioperative period of kidney transplantation can be associated with various factors. These include metabolic disorders (acidosis, hyperchloremia), use of anticoagulants before surgery, previous hemorrhagic tendency, intraoperative events (duration of surgery, hypothermia, anemia, variations in blood pressure), surgical technical variables (vascular anatomy, anastomosis sites, and suture technique), and factors specific to the recipient/donor pairing (etiology of CKD, donor/recipient size ratio, length of dialysis therapy prior to transplantation, type of donor) [4, 9, 10, 13–16].

Currently, there is no established consensus on how to characterize severe bleeding in children undergoing kidney transplantation, which makes comparison between studies difficult. Based on the clinical experience of our group, we hypothesized that hemorrhagic complications in the perioperative period of kidney transplantation may be more frequent and more severe than suggested by the limited existing literature.

This study aimed to characterize severe perioperative hemorrhage and determine its frequency, risk factors, and consequences in pediatric kidney transplantation.

## Methods

This retrospective cohort study evaluated data collected from the physical and electronic medical records of children and adolescents who received their first transplant at Hospital Samaritano de São Paulo between April 2008 and September 2015. Patients with combined liver and kidney transplants were excluded, as were patients who underwent kidney retransplantation. The project was approved by the Research Ethics Committees of Federal University of São Paulo (UNIFESP) and of Hospital Samaritano (Number 4.848.464).

Given that there is no clear definition in the literature for severe hemorrhage, the first step in analyzing the data in this research was to establish criteria to define this condition, based on information from the records of the patients included in the study. After establishing the criteria for defining severe hemorrhage, we identified associated risk factors and subsequently evaluated its impact on pediatric kidney transplant outcomes.

To distinguish children with severe bleeding from those without this complication, we applied a k-means clustering approach. For each patient, bleeding volume and the volumes of transfused red blood cell concentrates, platelets, plasma, and cryoprecipitate from the time of surgery through postoperative day 3 were included in the clustering model. Volumes were expressed as mL/m^2^ of body surface area (BSA) and subsequently standardized as z-scores before inclusion in the analysis. All data were obtained from medical prescriptions and confirmed using records from the hemotherapy service.

The cluster analysis identified a group of children characterized by greater blood loss and/or higher transfusion requirements, which was classified as severe hemorrhage. Regardless of cluster allocation, patients were also classified as having severe hemorrhage if they experienced major bleeding-related clinical events until the tenth postoperative day, namely: a) reoperation due to transplant-related bleeding, b) use of recombinant activated factor VII (rFVIIa), or c) use of fibrinogen concentrate. These therapies were reserved for exceptional cases of severe bleeding in which conventional supportive measures were insufficient and were therefore considered markers of extreme hemorrhagic severity rather than routine perioperative management.

Therefore, severe hemorrhage was defined based on two non-exclusive criteria: (1) the patient belonged to the group identified by cluster analysis, and/or (2) they met criteria a, b, or c as previously described. Thirty-eight potential exposure variables for this outcome were examined as shown in Table 1.

**Table 1:** Potential risk variables for severe hemorrhage

Recipient clinical variables	
• Sex: male or female	
• Weight (kg)	
• Height (cm)	
• Etiology of kidney disease: CAKUT, FSGS, other glomerulopathies, congenital/hereditary diseases, systemic diseases, undetermined, or others	
• Treatment before transplantation: conservative treatment, peritoneal dialysis, or hemodialysis	
• Length of renal replacement therapy (months)	
• Mean arterial pressure at the end of surgery (mmHg)	
Donor clinical variables	
• Type of donor: living or deceased	
• Donor age (years)	
• Kidney graft dose in g/m² BSA (graft weight divided by recipient’s BMI)	
Surgical technical variables	
• Surgical time (min): from skin incision to skin closure	
• Cold ischemia time (hours): deceased donors only	
• Axillary temperature at the end of surgery (°C)	
• Intraoperative saline volume (mL/kg)	
• Preoperative anticoagulant use: yes or no	
• Donated kidney: right or left	
• Vascular anatomy: number of arteries and veins	
• Anastomosis sites: vessels used for arterial and venous anastomoses	
• Suture technique: interrupted, continuous, or continuous-interrupted	
Laboratory variables	
• Bicarbonate (mmol/L): preoperative and immediate postoperative periods	
• Chloride (mmol/L): preoperative and immediate postoperative periods	
• Ionized calcium (mmol/L): preoperative and immediate postoperative periods	
• Urea (mg/dL): preoperative and immediate postoperative periods	
• Hemoglobin (g/dL): preoperative, intraoperative, and immediate postoperative periods	
• Platelet count (×10³/μL): preoperative and immediate postoperative periods	
• APTT (seconds): preoperative and immediate postoperative periods • Preoperative INR (unitless)	

*CAKUT* congenital anomalies of the kidney and urinary tract; *FSGS* focal segmental glomerulosclerosis; *APTT* activated partial thromboplastin time; *INR* international normalized ratio

Subsequently, we analyzed the consequences of severe hemorrhage on the following kidney transplant outcomes: the occurrence of delayed renal function, defined as the need for dialysis in the first week after transplantation, hospitalization length during transplantation, estimated glomerular filtration rate one year post-transplantation, calculated using the modified Schwartz formula [17], and five-year graft and recipient survival.

Statistical data analysis was performed using R language version 4.4.1 (Copyright (C) 2024 The R Foundation for Statistical Computing) and RStudio software version 2024.09.0 + 375 (Copyright (C) 2024 by Posit Software, PBC). The median and interquartile range were used to describe the quantitative variables, while the qualitative variables were represented in the form of frequency tables. To identify patterns of severe hemorrhage, a cluster analysis was performed using the k-means algorithm. The model included standardized values (z-score) of the volume of bleeding (mL/m^2^BSA) from surgery to the third postoperative day, in addition to the z-scores of the volumes of blood components administered (red blood cell concentrate, fresh frozen plasma, platelet concentrate, and cryoprecipitate), thus allowing the identification of natural subgroups of patients with distinct transfusion and bleeding characteristics.

To study the risk factors associated with hemorrhage, unadjusted logistic regression analyses were developed, including the individual's group (severe bleeding present versus absent) as a binary outcome variable and each of the candidate exposure variables, one by one. In the final model, variables whose association had biological plausibility and a statistically significant association with the outcome were chosen.

In addition, to test the consequences of severe hemorrhage on transplant outcomes, survival analyses (for grafts and recipients) were developed using the Kaplan–Meier survival curves and groups were compared using the log-rank test. Finally, Fisher's exact test was used to compare proportions, and the Mann–Whitney U test was used for numerical variables. For all tests, the limit of 5% (α < 0.05) was adopted to reject the null hypothesis.

## Results

**Fig. 1:**
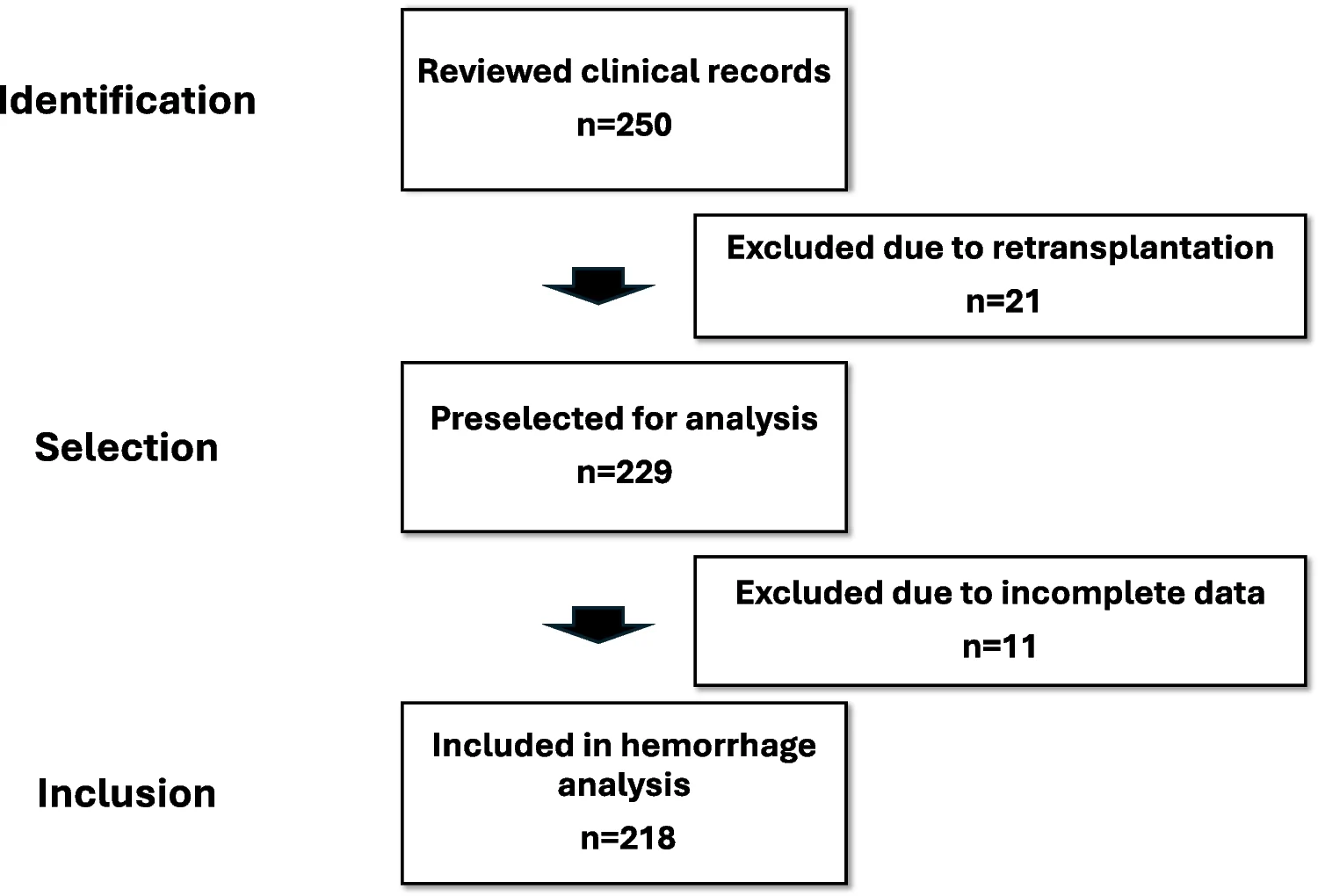
Sample selection process flowchart

During the study period, 250 pediatric kidney transplants were performed. Of these, 21 patients were excluded because they had undergone kidney retransplantation and 11 because of insufficient data in their clinical records, leaving 218 patients who formed the sample for this study. Figure 1 summarizes the patient selection process for the study.

Regarding postoperative immunosuppression, most patients received corticosteroids, tacrolimus, and azathioprine. Demographic, clinical and transplant characteristics of the study population are presented in Table 2.

**Table 2:** Demographic, clinical and transplant data of the study sample

Variable	General *n*=218
Male	139 (64%)
Recipient’s age (years)	7.9 (4.3–12.9)
Recipient's weight (kg)	19.6 (13.8–31.6)
Recipient's height (cm)	112.5 (92.0–137.0)
Cause of kidney disease	
CAKUT	112 (51%)
FSGS	32 (15%)
Glomerulopathies	13 (6%)
Congenital/hereditary	25 (11%)
Systemic disease	13 (6%)
Unknown disease	14 (7%)
Other causes	9 (4%)
Treatment before transplant	
Hemodialysis	147 (67%)
Peritoneal dialysis	49 (23%)
Conservative treatment	22 (10%)
RRT time (months)	18.0 (9.2–35.0)
Mean BP at the end of surgery	84 (71–96)
Living donor	42 (19%)
Deceased donor’s age (years)	12.0 (6.0–16.0)
Live donor's age (years)	37.5 (32.0–42.8)
Graft dose (g/m²BSA)	164 (107–246)
Surgical time (min)	158 (130–190)
CIT (hours) - deceased donors only	23.4 (19.4–27.2)
Axillary temperature at the end of surgery (ºC)	36.0 (36.0–36.0)
IO SS volume (ml/kg)	51.0 (36.0–71.0)
Thrombosis after Tx	5 (2.3%)
Loss of Tx	40 (18%)
Death of the recipient	2 (1.0%)

Numerical variables expressed as median (IQR) and qualitative variables expressed as frequency and percentage; *BSA* body surface area; *CAKUT* malformations of the kidneys or urinary tract; *FSGS* focal segmental glomerulosclerosis; *PO* postoperative; *min* minutes; *IO* intraoperative; *Tx* transplant; *SS* saline solution; *CIT* cold ischemia time

The k-means algorithm implemented in the development of the clustering model resulted in the discrimination of two groups: Group 1, with 12 cases including children with greater blood loss and/or required increased volumes of blood components, and Group 2 with 206 cases with lower volumes of bleeding and infusion of blood components. These groups are depicted in the supplement Figure S1.

In addition to the 12 patients characterized by the clustering technique, other patients were classified as having severe hemorrhage due to transplant-related bleeding events, such as reoperation due to bleeding (5 cases) and/or the use of activated recombinant factor VII (7 cases), and/or fibrinogen (2 cases); however, most of these cases had already been captured by the clustering method, resulting in only four newly classified patients. Importantly, this situation occurred in patients whose bleeding-related clinical events took place very early in the postoperative period, before substantial bleeding or transfusion volume had accumulated over the first postoperative days. For this reason, they were not captured by the cluster-based definition alone and were therefore included as an additional criterion. Therefore, among all patients studied, 16 (7.3%) were characterized as having severe hemorrhage, while the remaining 202 patients comprised the group without the outcome.

**Fig. 2:**
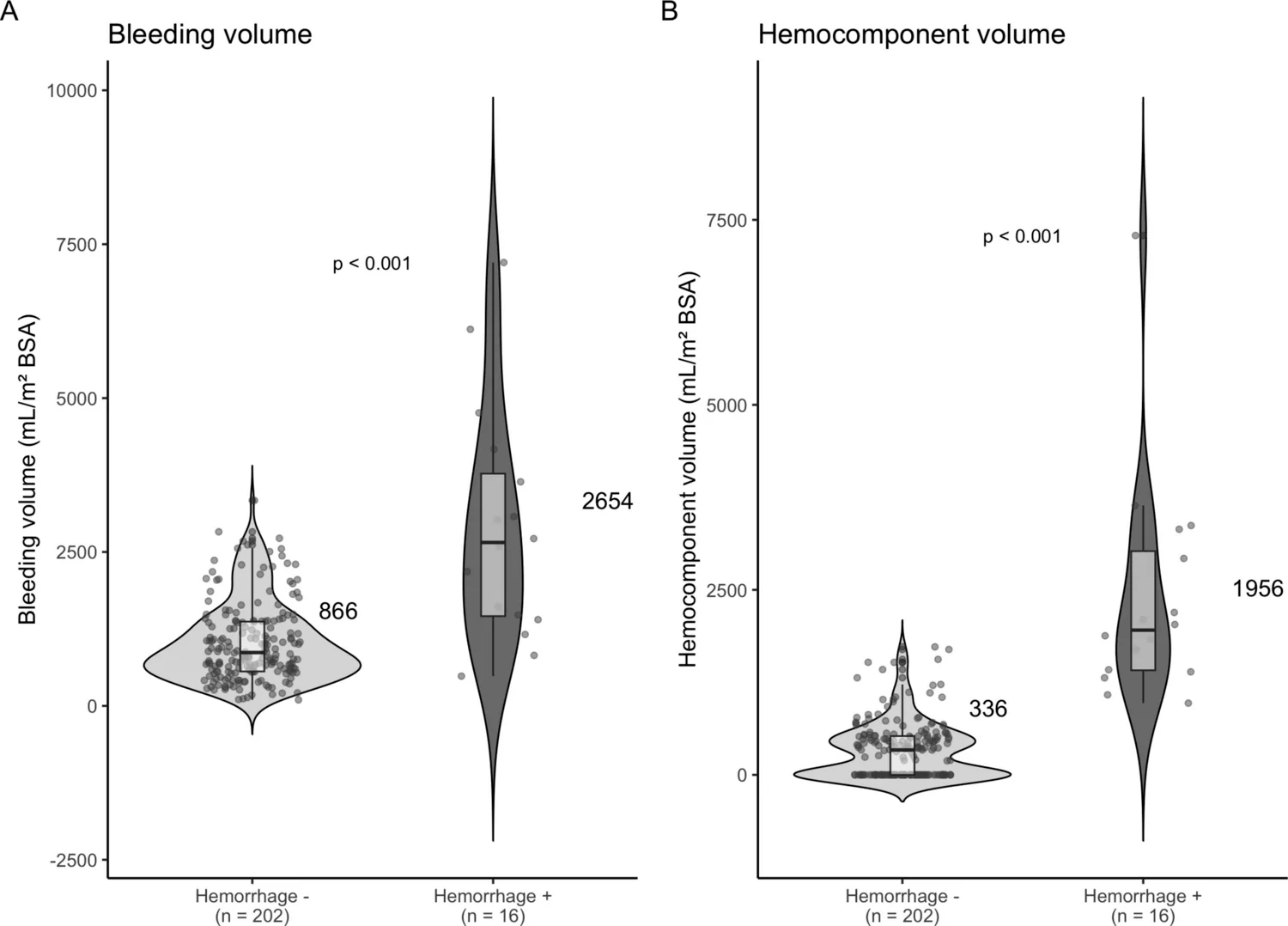
Bleed volume (**A**) and hemocomponent volume (**B**) from postoperative days d0 to d3 categorized by groups

To illustrate the identification capacity of the applied strategy, the median bleeding volume between the immediate postoperative phase and postoperative day 3 in the group with severe hemorrhage was 2,654 mL/m^2^BSA (IQR = 1,458–3,772), while in the group without severe hemorrhage this value was 866 mL/m^2^BSA (IQR = 559–1369), a statistically significant difference (*p* < 0.001). When expressed in clinically intuitive units, the cumulative bleeding volume from the day of surgery through postoperative day 3 corresponded to a median of 29.6 mL/kg/day (IQR 16.3–40.8) in those with severe hemorrhage and 8.4 mL/kg/day (IQR 5.1–13.0) in patients without severe hemorrhage. Similarly, the infused hemocomponent volume in the group with severe hemorrhage was 1,956 mL/m^2^BSA (IQR = 1,413–3,023), and 336 mL/m^2^BSA (IQR = 0–524) in the group without severe hemorrhage (*p* < 0.001). For reference, median BSA was 0.49 m^2^ (IQR, 0.48–0.66) in the group with severe hemorrhage and 0.77 m^2^ (IQR = 0.58–1.09) in the group without severe hemorrhage. Both volumes are depicted in Fig. 2.

Of the thirty-eight variables considered, acidosis, hyperchloremia, anemia, cold ischemia time, surgical time, sex, intraoperative saline solution volume, uremia, cause of kidney disease, temperature at the end of surgery, and all surgical technique-related variables evaluated, despite being previously cited as potential risk factors, were not associated with severe hemorrhage in the present study.

**Fig. 3:**
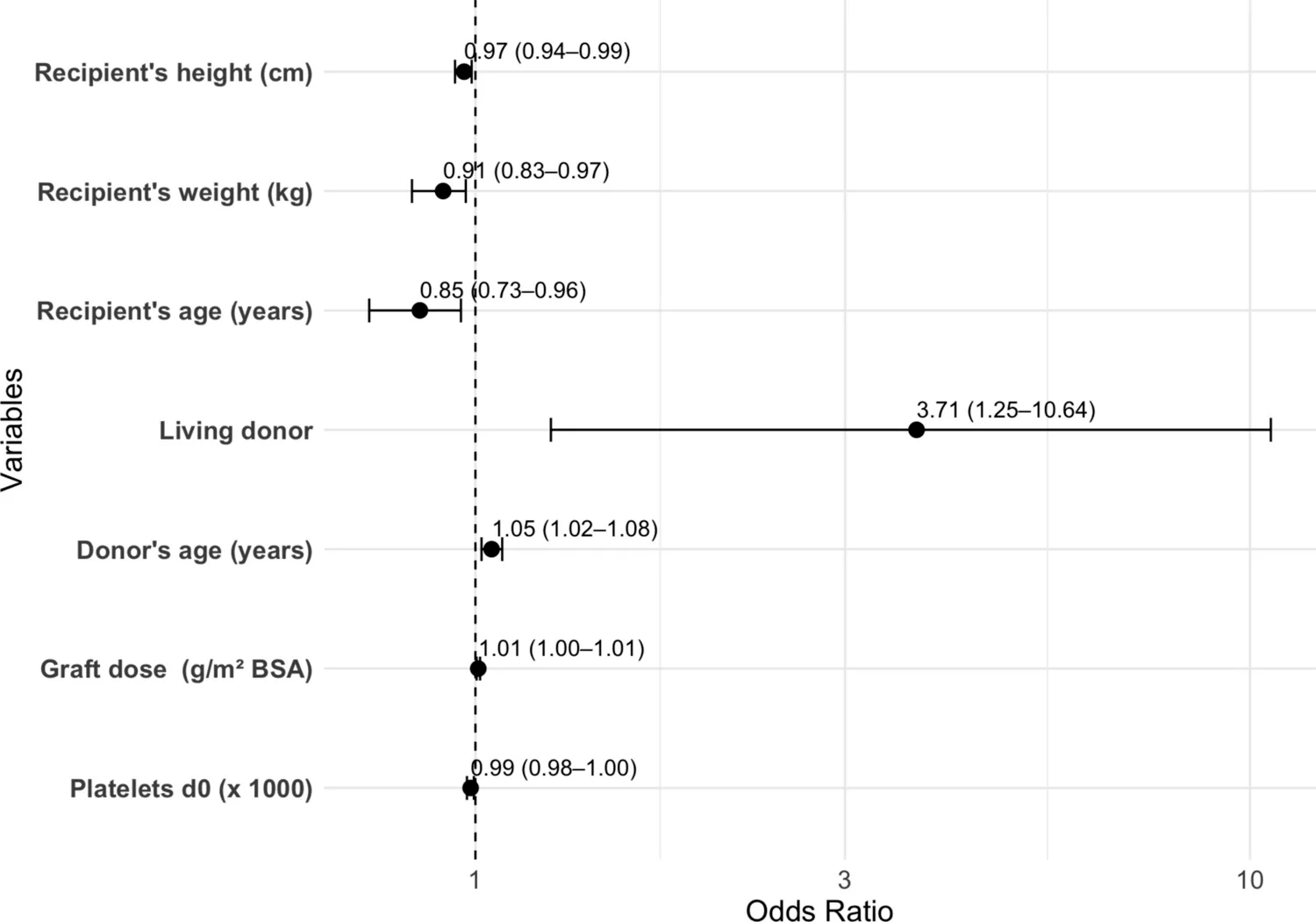
Variables with significant association with severe hemorrhage in simple logistic models

Seven variables were statistically associated with the study outcome in the unadjusted logistic regression analyses. Younger age, lower weight, and height were associated with a higher risk of bleeding. Additionally, kidney recipients who received organs from older donors, from living donors, and a higher graft dose also had a higher risk of hemorrhage. Among the laboratory risk factors for bleeding, the platelet count on the first postoperative day was the only variable that was significantly associated with the occurrence of severe hemorrhage. The full comparative analysis of all variables between the two groups is provided in Supplementary Table S1, while Table 3 presents only the factors that were significantly associated with severe hemorrhage. These results are illustrated in Fig. 3.

**Table 3:** Significant risk factors and outcomes associated with severe hemorrhage between groups

Significant risk factors	Group 1 *n*=16	Group 2 *n*=202	*p*
Recipient’s age (years)	2.8 (2.2–8.0)	8.5 (4.5–13.0)	0.003
Recipient’s weight (kg)	11.2 (10.4–14.6)	19.2 (13.1–30.0)	0.001
Recipient’s height (cm)	85.5 (81.5–106)	115 (94–137)	0.002
Living donor	7 (44%)	35 (17%)	0.017
Donor's age (years)	33.5 (12.5–44.8)	14.0 (8.0-28.0)	0.014
Graft dose (g/m²BSA)	293 (177–329)	159 (104–239)	0.002
Platelets d1 (× 10^3^)	150 (91–163)	197 (155–252)	0.002
Significant outcomes			
Graft survival (5 years)	62.5% (42.8–91.4)	91.5% (87.6–95.6)	< 0.001
LOS (days)	10 (8–15)	7 (6–11)	0.03

*LOS* Length of hospital stay

A multiple logistic model was not developed because the limited number of observed outcomes would not allow the inclusion of many exposure variables, which would constrain the ability to examine several risk factors within the same model.

**Fig. 4:**
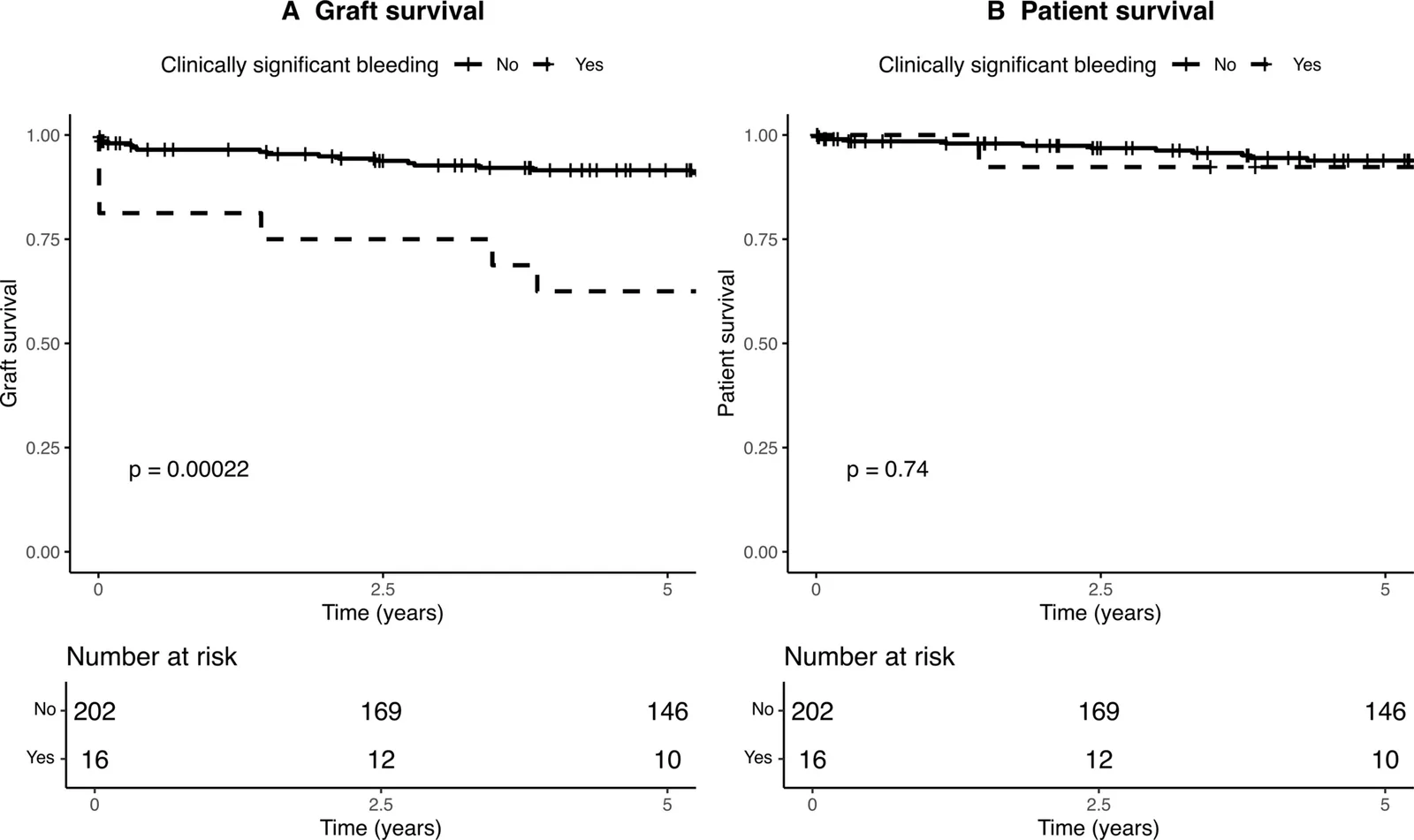
Five-year graft (**A**), and patient’s survival (**B**) by groups

No significant between-group difference was observed in the need for dialysis within the first week after transplantation, which occurred in four children (25%) with severe hemorrhage and in 55 (27%) without it (*p* = 1.0). In contrast, median hospital stay was longer in those with severe hemorrhage: 10 days (IQR, 8–15) versus 7 days (IQR, 6–11) (*p* = 0.003). Estimated glomerular filtration rate during the first postoperative year was similar between groups, with median values of 66 mL/min/1.73 m^2^ BSA (IQR, 54–83) and 70 mL/min/1.73 m^2^ BSA (IQR, 57–83), respectively (p = 0.666). Five-year graft survival was lower among affected children (62.5% vs. 91.5%; p < 0.001), whereas patient mortality did not differ between groups. Graft and patient survival curves are shown in Fig. 4, and Table 3 presents only the outcomes significantly associated with this complication. Two patients in the study cohort died, and neither death was related to hemorrhagic complications.

## Discussion

The main finding of this study was the characterization of severe hemorrhage and its frequency based on the data from our study group. Severe hemorrhage was defined using a composite, data-driven approach that incorporated postoperative bleeding volume, transfusion requirements, and major bleeding-related clinical events, such as reoperation or the use of fibrinogen and/or recombinant factor VIIa. Therefore, rather than identifying independent predictors of severe hemorrhage, our analyses helped to describe the clinical profile and practical correlates of this complication in the perioperative period of pediatric kidney transplantation. In our sample, the frequency of severe hemorrhage was 7.3%, which is higher than the 4.1% to 4.5% range reported in the literature [7, 8]. The higher observed rate of severe hemorrhage in our study is largely due to the definition we used. Instead of relying solely on traditional clinical criteria—such as the need for surgical re-exploration—we employed a cluster analysis approach. This method grouped patients based on greater postoperative bleeding volumes or higher utilization of blood products. As a result, our definition is broader and data-driven, which may not align directly with the narrower criteria commonly applied in other pediatric transplant programs. The previous studies may have limitations because they used definitions of severe hemorrhage applicable to adult populations without underlying kidney disease.

To translate these findings into practical clinical terms, two relevant patterns emerged. First, cumulative bleeding below approximately 1,400 mL/m^2^ from Day 0 to Day 3 (approximately 350 mL/m^2^/day or 8.4 mL/kg/day), together with transfusion of no more than one unit of packed red blood cells, was associated with a low probability of classification as severe hemorrhage. Conversely, the need for plasma, cryoprecipitate, or platelet transfusion, even at low volumes, was strongly associated with this classification, suggesting that early use of these hemostatic components may serve as a practical marker of clinically relevant bleeding.

Beyond its frequency, we next examined the surgical characteristics of severe hemorrhage in this cohort and none of the variables related to the surgical procedure were statistically associated with the outcome. Moreover, among the five patients who required reoperation, only one had an identifiable bleeding focus, whereas most exhibited diffuse coagulopathy with hematoma formation and consumption of coagulation factors. Some cases required more than one surgical intervention or the use of hemostatic packing, and one patient required temporary abdominal closure using a Bogota bag. Collectively, these findings indicate the lack of a detectable association between surgical/anatomical factors and severe hemorrhage in this cohort.

Severe hemorrhage is more commonly observed in the initial days following surgery, as reported in previous studies involving adult patients [9, 18]. In our study, we also noted a higher incidence of this complication on the first postoperative day, underscoring the importance of vigilant monitoring for hemorrhagic events during the first 24 h.

Previous publications point to uremia, the duration of dialysis treatment prior to transplantation, and coagulation disorders associated with CKD as risk factors for severe bleeding [9, 19, 20]. Additionally, metabolic disorders frequently observed in patients with CKD, such as acidosis, can affect the coagulation cascade and favor severe hemorrhage, thereby compromising graft survival [21]. In our study, we also evaluated these factors in patients both during the preoperative period and in the first three postoperative days. However, we found no association between these factors and severe bleeding. This may be due to the fact that our sample comprised pediatric patients in whom the effect of each of these factors may differ from adults.

Studies in adults have indicated that kidney transplants from living donors (OR 0.30 [95% CI: 0.16, 0.55]) and higher recipient body mass index (BMI) (OR 0.54 [95% CI: 0.30, 0.99]) are linked to a reduced risk of bleeding compared to transplants from deceased donors and with lower recipient BMI [9, 22]. Furthermore, chronic use of anticoagulants in the preoperative period was associated with an increased risk of bleeding, but without statistical significance (OR 1.75 [95% CI: 0.52, 5.88]) [9, 22]. In our study, we observed a significant association between donor and recipient size disproportion in pediatric kidney transplantation and the occurrence of severe hemorrhage. In younger pediatric recipients, the donor graft is frequently relatively large in relation to the recipient’s body size. This anatomical disproportion may add technical complexity to the procedure and has been linked to increased perioperative transfusion requirements in previous studies. In a Canadian cohort of 188 pediatric kidney transplant recipients, oversized grafts were associated with significantly higher intraoperative blood loss, attributed to increased bleeding, greater blood sequestration within the graft, and the need for more extensive surgical dissection to accommodate the larger transplanted kidney [19]. Another potential explanation, although speculative, is that larger grafts may retain greater volumes of the preservation solution containing heparin, which could theoretically contribute to bleeding in small recipients.

In Brazil, pediatric candidates are prioritized for kidneys from deceased donors younger than 18 years. As a result, children receiving kidneys from deceased donors are more likely to receive organs from pediatric donors, whereas living donors are necessarily adults. Therefore, living donor transplantation may involve greater donor–recipient body size disparity. We speculate that this size mismatch, rather than living donation itself, may partly explain the association between living donation and hemorrhagic complications observed in our sample. In addition, we observed that the increased risk of bleeding in recipients with a lower BMI was similar to that described in studies conducted with adults, suggesting that a closer match in body size between donor and recipient may contribute to a better outcome.

Additionally, chronic anticoagulant use was not associated with severe hemorrhage in our study. The low frequency of their use in our sample (3/218) may explain the failure to find this association, which is biologically plausible. Notably, only one patient received postoperative heparin, without any associated hemorrhagic complications.

Platelet count at the end of surgery was another factor associated with a higher risk of severe hemorrhage. In our sample, an increase of 1,000 platelets per microliter of blood was associated with a 1% reduction in the risk of this complication. However, a causal relationship cannot be established, as the observed decrease in platelet count may also reflect a consequence of active bleeding due to platelet consumption during the coagulation process.

In our sample, patients with severe hemorrhage had a lower probability of five-year graft survival than those without severe bleeding. This event may be related to acute kidney injury caused by severe hemorrhage in the perioperative period or to early graft loss as a result of hemorrhage. In our study there was no evidence of difference in patient mortality, unlike some studies in adults [9]. Hachem et al. evaluated 59 patients and found that the cumulative probability of kidney graft loss and/or death was higher in patients with hemorrhage in the first three postoperative days compared to the group without hemorrhage (HR 1.62 [95% CI: 1.01, 2.60]) [9], which would be justified by the comorbidities associated with adult/elderly patients that were absent in the pediatric age group.

Finally, severe hemorrhage was associated with prolonged hospital stay in our cohort. Nonetheless, information on the length of hospital stay after pediatric kidney transplantation remains scarce. This outcome is relevant both for patient quality of life and for its economic impact on the healthcare system [20, 23].

This study has several limitations. Its retrospective, single-center design limits causal inference and generalizability, and it was based on a convenience sample using relatively old data collected between 2008 and 2015, as those were the years for which data were most readily available. Also, in 2016 the hospital underwent a major institutional transition, changing from a philanthropic to a for-profit hospital, with substantial changes in its structure and processes. Moreover, the limited number of events precluded us from performing multivariable logistic regression to identify risk factors for postoperative hemorrhage. However, to our knowledge, this is the largest study to date that specifically focuses on severe postoperative hemorrhage in pediatric kidney transplant recipients. In addition, most previous studies in this population have relied on adult-based criteria to define severe hemorrhage. Finally, we applied modern clustering techniques to identify distinct clinical profiles potentially associated with this outcome [24].

In conclusion, severe hemorrhage after pediatric kidney transplantation performed in our center occurred more frequently than previously reported, particularly during the first postoperative day, and was significantly associated with donor–recipient size disproportion. These findings underscore significant consequences, particularly concerning reduced graft survival. The primary risk group consists of smaller recipients receiving relatively larger grafts. Given the limited evidence available for this age group, our results contribute valuable information to the literature, although validation in independent cohorts is necessary.

## Supplementary Information

Below is the link to the electronic supplementary material.

Graphical abstract (PPTX 824 KB)

Supplementary file2 (DOCX 59 KB)

## Data Availability

The datasets generated and/or analysed during the current study are available from the corresponding author on reasonable request.

## Abbreviations

BSA: Body surface area
CKD: Chronic kidney disease
Hb: Hemoglobin
ISTH: The International Society on Thrombosis and Haemostasis
