# Supplementary material for: Perioperative severe hemorrhage in pediatric kidney transplantation: frequency, risk factors, and outcomes
Source: Pediatr Nephrol. 2026 Jun 6;41(11):4027–36. doi: 10.1007/s00467-026-07391-7 (PMC13612639; doi:10.1007/s00467-026-07391-7)
Supplement: Supplementary file 1 [file 467_2026_7391_MOESM2_ESM.docx]

Figure S1 – Graphical representation of the results of the clustering technique

Figure S1 represents the results of the five volumes considered in the model (bleeding and infusion of each blood component) on two axes to simplify visualization while maintaining the most relevant information from the original variables. Assessing the contribution of each volume in our model, Dimension 1 is the most important, with relatively balanced contributions from all variables (platelets, cryoprecipitate, plasma, red blood cells and bleeding), while in Dimension 2 the bleeding variable is the one that contributed the most.

Table S1. Comparison of risk factors for severe hemorrhage between groups

| Variable | Group 1 n=16 | Group 2 n=202 | *p* |
| --- | --- | --- | --- |
| Sex (Male) | 9 (56.3) | 130 (64.3) | 0.592 |
| Recipient’s age (years) | 2.8 (2.2–8.0) | 8.5 (4.5–13.0) | 0.003 |
| Recipient’s weight (kg) | 11.2 (10.4–14.6) | 19.2 (13.1–30.0) | 0.001 |
| Recipient’s height (cm) | 85.5 (81.5–106) | 115 (94–137) | 0.002 |
| Cause of kidney disease |  |  | 0.690 |
| CAKUT | 8 (50%) | 104 (52%) |  |
| FSGS | 2 (13%) | 30 (15%) |  |
| Glomerulopathies | 2 (12%) | 11 (5%) |  |
| Congenital/hereditary | 1 (6%) | 24 (12%) |  |
| Systemic disease | 1 (6%) | 12 (6%) |  |
| Unknown disease | 2 (13%) | 12 (6%) |  |
| Other causes | 0 | 9 (4%) |  |
| Treatment before transplant |  |  | 0.507 |
| Hemodialysis | 12 (75%) | 135 (67%) |  |
| Peritoneal dialysis | 4 (25%) | 45 (22%) |  |
| Conservative treatment | 0 | 22 (11%) |  |
| KRT time (months) | 12 (8–24) | 18 (10–36) | 0.113 |
| Mean BP | 78 (62–93) | 84 (72–96) | 0.256 |
| Living donor | 7 (44%) | 35 (17%) | 0.017 |
| Donor's age (years) | 33.5 (12.5–44.8) | 14.0 (8.0-28.0) | 0.014 |
| Graft dose (g/m²BSA) | 293 (177–329) | 159 (104–239) | 0.002 |
| Surgical time (min) | 175 (144–199) | 155 (130–190) | 0.212 |
| Deceased donors CIT (hours) | 24 (19–27) | 22 (19–28) | 0.636 |
| Axillary temperature (ºC) | 36 (36–36) | 36 (36–36) | 0.132 |
| IO SS volume (ml/kg) | 63 (38–93) | 50 (35–70) | 0.277 |
| Preoperative anticoagulants | 3 (1.5%) | 0 | 1,000 |
| Donated kidney (left) | 71% | 61% | 0.574 |
| Number of renal arteries (> 1) | 0% | 19% | 0.385 |
| Number of renal veins (> 1) | 0% | 3% | 1 |
| Arterial anastomosis (Aorta) | 54% | 36% | 0.446 |
| Venous anastomosis (Cava) | 71% | 56% | 0.237 |
| Continuous-interrupted suture | 89% | 92% | 0.562 |
| Bicarbonate d0 | 25 (21–27) | 22 (20–26) | 0.267 |
| Bicarbonate d1 | 22 (20–24) | 21 (19–23) | 0.110 |
| Chloride d0 | 101 (98–104) | 102 (99–106) | 0.778 |
| Chloride d1 | 104 (101–104) | 104 (100–108) | 0.665 |
| Ionized calcium d0 | 1.22 (1.18–1.25) | 1.19 (1.10–1.25) | 0.350 |
| Ionized calcium d1 | 1.12 (1.06–1.25) | 1.17 (1.09–1.23) | 0.649 |
| Urea d0 | 68 (45–99) | 96 (62–130) | 0.050 |
| Urea d1 | 67 (44–80) | 83 (60–114) | 0.082 |
| Hemoglobin d0 | 10.6 (9.2–11.8) | 11.2 (9.7–12.6) | 0.312 |
| Hemoglobin d1 | 11.0 (9.3–12.4) | 10.5 (9.5–12.0) | 0.853 |
| Platelets d0 (× 10^3^) | 224 (184–287) | 230 (195–303) | 0.482 |
| Platelets d1 (× 10^3^) | 150 (91–163) | 197 (155–252) | 0.002 |
| APTT d0 | 38.6 (34.2–51.7) | 38.2 (34.7–41.8) | 0.789 |
| APTT d1 | 45.2 (39.0–49.7) | 38.2 (33.6–44.1) | 0.128 |
| INR d0 | 1.00 (0.97–1.10) | 1.00 (0.96–1.10) | 0.966 |

Numerical variables expressed as median (IQR) and qualitative variables expressed as frequency and percentage, Group 1 = children with severe hemorrhage, Group 2 = children without severe hemorrhage, CAKUT = malformations of the kidneys or urinary tract, FSGS = focal segmental glomerulosclerosis, KRT = kidney replacement therapy, SS = saline solution, IO = intraoperative, d0 = preoperative period, d1 = immediate postoperative period, Tx = transplant, BSA = body surface area
